# Dysregulated proteolytic cascades in Netherton syndrome: from molecular pathology to preclinical drug testing

**DOI:** 10.1002/path.70018

**Published:** 2026-01-09

**Authors:** Eleni Zingkou, Evangelos Bisyris, Georgios Pampalakis, Georgia Sotiropoulou

**Affiliations:** ^1^ Department of Pharmacy, School of Health Sciences University of Patras Rion‐Patras Greece; ^2^ Laboratory of Pharmacology, School of Pharmacy Aristotle University of Thessaloniki Thessaloniki Greece

**Keywords:** Netherton syndrome, proteolytic cascades, knockout models, transgenic models, organotypic cultures, kallikrein‐related peptidases (KLKs)

## Abstract

Netherton syndrome (NS) is a rare, severe, and often life‐threatening disease for which current therapeutic approaches are limited and show variable effectiveness. NS is characterized by excessive epidermal desquamation that results in a highly defective epidermal barrier, constitutive skin inflammation, allergies, and hair abnormalities. NS develops due to loss‐of‐function mutations in the *SPINK5* gene, which encodes the LEKTI inhibitor that regulates KLK proteases (KLK5, KLK6, KLK7, KLK13, and KLK14). These findings indicate that dysregulation of proteolytic networks underlies the extensive skin shedding and inflammation characteristic of NS. *Spink5* ^−/−^ mice recapitulate the major features of the human disease but exhibit neonatal lethality. Several double‐ and triple‐knockout models have been generated to rescue the lethal NS phenotype, and have proved instrumental in studies aiming to elucidate the biological pathways involved in NS, and to identify and validate potential targets for drug development. These studies have established that inhibition of excessive KLK protease activity in LEKTI‐deficient epidermis can reverse the cutaneous manifestations of NS. In particular, ablation of KLK5 results in a marked therapeutic response, although KLK7 or TNFα must also be inhibited to rescue the most severe (lethal) form of NS. Murine models have also been essential in proving or disproving putative pathways and/or therapeutic targets proposed from *in vitro* studies or patient case studies. Collectively, these models have provided a deeper understanding of the epidermal proteolytic cascades involved in NS pathology and in normal skin renewal. Moreover, these models offer a platform in which disease‐specific candidate therapeutics can be tested and preclinically validated. © 2026 The Author(s). *The Journal of Pathology* published by John Wiley & Sons Ltd on behalf of The Pathological Society of Great Britain and Ireland.

## Introduction

**Figure 1:**
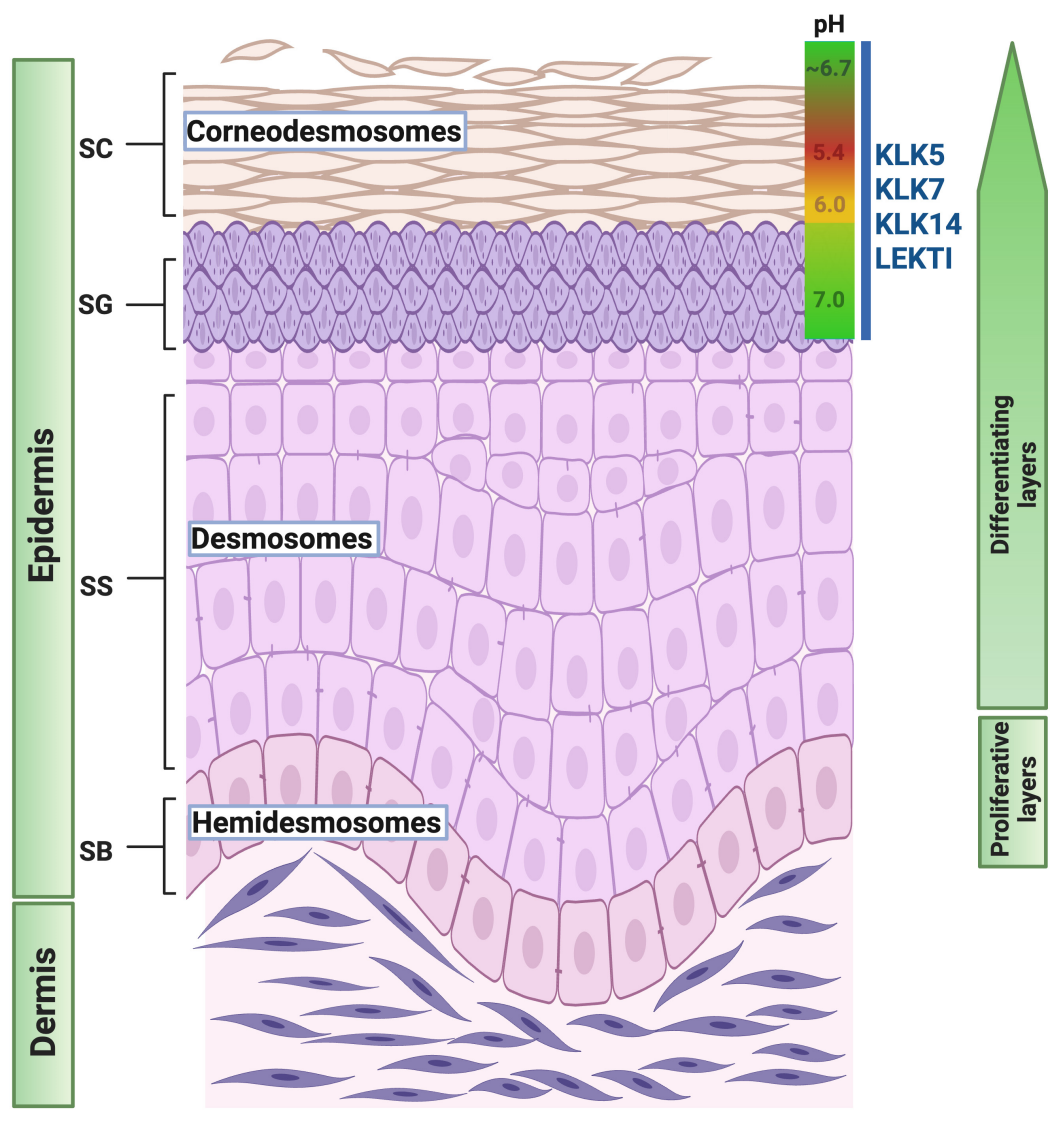
Schematic structure of human skin. Created in BioRender. Zingkou E (2025) https://BioRender.com/1sgwl3x

Skin is the largest organ of the human body and is composed of the hypodermis, dermis, and epidermis. The epidermis is a stratified epithelium that encompasses the outer skin layer and forms the physical barrier against the environment. It mostly contains keratinocytes and, to a lesser degree, melanocytes that synthesize melanin, as well as Langerhans cells that act as antigen‐presenting cells [1] and Merkel cells that function as touch receptors [2]. The epidermis is further subdivided into four different layers, each one comprising keratinocytes at different differentiation states. The innermost layer bound to the basal membrane, the stratum basale (SB), contains the least differentiated cells and some keratinocyte stem cells. The next layer is the stratum spinosum (SS), followed by the stratum granulosum (SG), which is the last epidermal living layer, preceding the stratum corneum (SC). The SC is composed of multiple layers of terminally differentiated keratinocytes, termed corneocytes, filled with keratins and lacking nuclei (Figure 1). In tissues such as the palms and soles, an additional epidermal layer, the stratum lucidum (SL), is found between the SG and the SC. The SC serves as the main epidermal barrier, preventing water loss, entry of chemicals and microorganisms, and provides resistance to mechanical stress [3, 4]. The corneocytes in the SC are held together by intercellular junctions called corneodesmosomes, which are specialized desmosomes made of desmoglein 1, desmocolin 1, and corneodesmosin. The SC resembles a brick‐and‐mortar pattern, where the bricks are the corneocytes and the mortar is the lipids [5]. The structure of human and mouse skin is similar but differs mainly in thickness and in the number of hosted hairs. Specifically, in human skin, the epidermis is thicker but has a significantly lower number of hair follicles.

## Renewal of the epidermis is regulated by a KLK‐mediated proteolytic cascade

Physiological renewal of the epidermis depends on a tightly regulated proteolytic cascade mediated by kallikrein‐related peptidases (KLKs), whose activities are regulated primarily by the endogenous protease inhibitor LEKTI (lympho‐epithelial Kazal‐type inhibitor) and by the epidermal pH gradient [6]. In brief, keratinocytes in the SG secrete KLK zymogens (proKLKs), mainly proKLK5, proKLK7, and proKLK14, and LEKTI‐derived inhibitory peptides [7, 8]. LEKTI, encoded by *SPINK5*, is a 15‐domain protease inhibitor, which is proteolytically processed to shorter peptides with protease inhibitory activity [9]. Once secreted, proKLK5 is autoactivated at the SG, where the pH is neutral (approximately pH 7.2), but its activity is rapidly quenched by LEKTI. Similarly, any activated proKLK7 and proKLK14 will be rapidly quenched by LEKTI. As the complexes diffuse to the outer SC, where the pH is acidic (approximately pH 5.5) due to the acid mantle, dissociation of the KLK5–LEKTI complex occurs. Active KLK5 protease then initiates the KLK cascade, generating the proteolytic activities required for cleavage of corneodesmosomes, leading to corneocyte shedding (epidermal desquamation).

**Figure 2:**
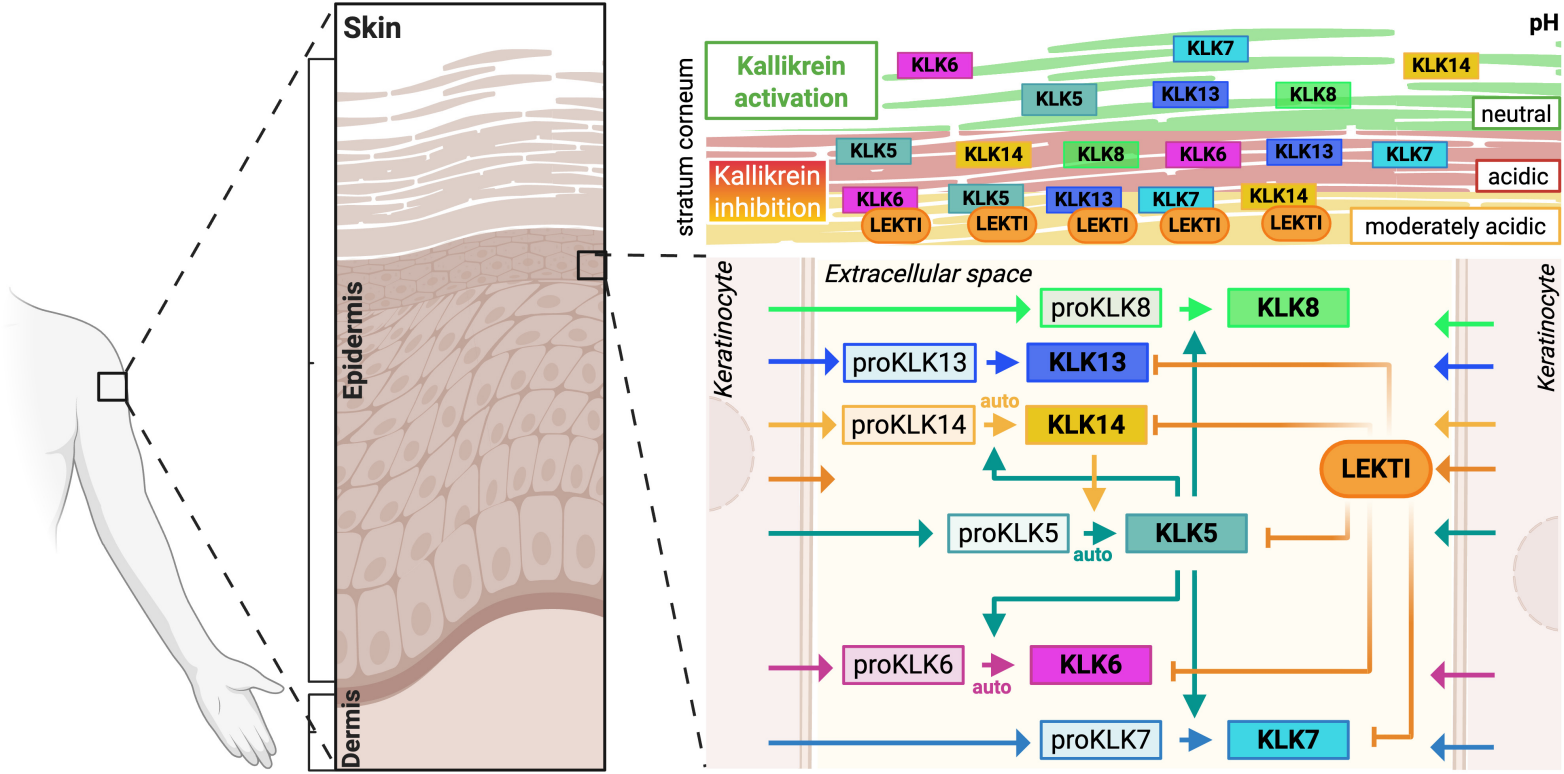
The epidermal KLK cascade and its regulation by the three epidermal pH zones. Created in BioRender. Zingkou E (2025) https://BioRender.com/96s7daw

A recent study using transgenic (*Tg*) mice encoding fusion proteins that display pH‐dependent fluorescence has provided a valuable tool for intravital pH imaging and refinement of the epidermal pH gradient. The study identified three distinct sublayers of pH or pH zones in the SC: an inner zone displaying a moderately acidic pH of approximately 6; a middle zone corresponding to the acid mantle with a pH of approximately 5.5; and an outer surface displaying almost neutral pH, due to ‘neutralization’ by the skin microbiota [10]. Based on these recent findings, the KLK‐mediated epidermal desquamation cascade has been refined. Specifically, in the lower pH zone, LEKTI peptides form complexes with active KLK proteases, quenching KLK activities. Dissociation of LEKTI takes place in the middle acidic zone, although the low pH still suppresses the catalytic activity of KLKs. This allows KLKs to remain in an inactive state in the middle‐acidic pH SC zone but become activated in the upper‐nearly neutral pH SC zone, where they mediate desquamation by proteolytic cleavage of corneodesmosomes (Figure 2).

## The *SPINK5* gene is linked to Netherton syndrome and atopic dermatitis

Netherton syndrome (NS) is a rare (1:200,000 births) but often life‐threatening disease characterized by excessive epidermal desquamation, constitutive skin inflammation, allergies, and a hair shaft defect known as ‘bamboo hair’. NS is caused by inactivating mutations in *SPINK5* and is inherited in an autosomal recessive manner [11]. LEKTI deficiency results in unopposed proteolytic activities at the SG–SC boundary, leading to premature SC detachment from the underlying SG. In turn, this severe defect of the SC results in dehydration, persistent inflammation, and increased susceptibility to invasion by pathogens and allergens. In addition, *in vitro* experiments have shown that KLKs can cleave the human growth hormone. Notably, *KLK*s and *SPINK5* are co‐expressed in the pituitary gland. Therefore, in NS, increased KLK activity in the pituitary gland may result in reduced growth hormone availability resulting in reduced body size, another recognized clinical feature of NS [12].

NS patients suffer from severe atopy associated with increased expression of thymic stromal lymphopoietin (TSLP) [13], suggesting shared underlying molecular mechanisms between NS and atopic dermatitis (AD). Indeed, the LEKTI variant E420K (*SPINK5 G1258A*) has been linked to AD [14, 15, 16]. One study showed that maternal transmission of LEKTI E420K increases the risk of eczema, although it is not considered a major risk factor [17]. Furthermore, reduced levels of LEKTI were found in keratinocytes isolated from AD patients [18]. In addition, the polymorphism *SPINK5* SNP rs9325071 (A→G) has been associated with food allergies in infants [19], although other studies reported no association in certain populations [20]. Therefore, the association of *SPINK5* polymorphisms with AD remains uncertain and may be limited to certain populations. Recent studies have suggested that the NS molecular signature is more closely related to psoriasis than to AD [21]. Analysis of the molecular mechanisms governing NS and the development of NS mouse models may provide valuable tools for detailed investigation of the molecular pathology of psoriasis and the identification of new therapeutic targets.

## Development of the *Spink5* ^−/−^ mouse as a model for NS

Since the genetic basis of NS was identified [11], there has been an urgent need for an animal model, which can recapitulate human NS, to enable investigation of the molecular mechanisms, and to identify and validate new pharmacological targets. Between 2004 and 2005, three independent groups generated *Spink5* ^−/−^ mice using gene targeting technologies [22, 23, 24]. Specifically, Descargues *et al* [22] replaced the ATG translational start codon and the first four exons of the mouse *Spink5* gene with an inactivating cassette, while Hewett *et al* [23] introduced the R820X mutation, which mimics the E827X mutation found in NS patients. Homozygous *Spink5* ^*R820X/R820X*^ mice show diminished expression of *Spink5* mRNA likely due to nonsense‐mediated decay. Finally, Yang *et al* [24] deleted a 66.8‐kb region encompassing the entire *Spink5* gene. In all three models, mice homozygous for *Spink5* ablation recapitulated NS symptoms and died homogeneously within approximately 5 h from birth due to severe dehydration.

Notably, the lethal phenotype of *Spink5* ^−/−^ mice results from a complete absence of LEKTI protein. In humans, NS is categorized into mild, moderate, and severe forms. This variability arises because different mutations in *SPINK5* can lead to residual expression of LEKTI, which in turn accounts for the variation in disease severity [25].

**Figure 3:**
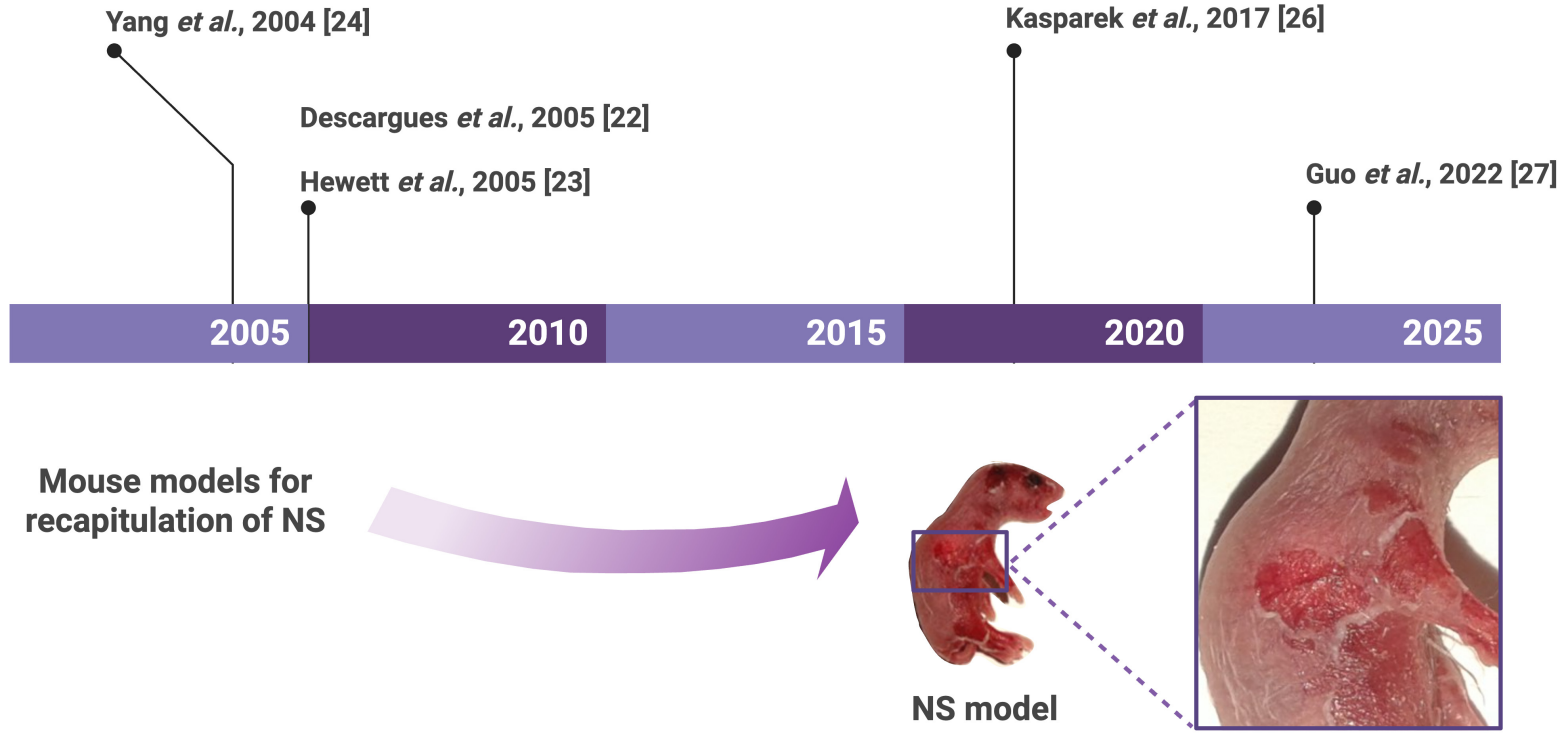
Timeline of the *Spink5* ^−/−^ mouse development and a representative image of the *Spink5* ^*R820X/R820X*^ mouse. Created in BioRender. Zingkou E (2025) https://BioRender.com/buf0pnm

Newer technologies based on transcription activator‐like effector nucleases (TALENs) have also been applied to generate *Spink5* ^−/−^ mice. TALENs were used to delete the TG dinucleotide sequence at positions 402–403 of the mouse *Spink5* gene to recapitulate the mutation p.A134X (398delTG) identified in the human *SPINK5* gene in NS patients [26]. Specifically, mRNAs encoding for two TALENs targeting the appropriate region within exon 5 were microinjected into C56BL6/N‐derived zygotes along with a single‐stranded oligonucleotide to introduce the desired deletion. The homozygous mice *Spink5* ^*A135X/A135X*^ died within 12 h from birth due to severe dehydration. Finally, CRISPR–Cas9 technology was applied for inactivation of *Spink5*. The Cas9 nuclease and the appropriate sgRNA targeting exon 3 of *Spink5* were microinjected into C57BL/6J zygotes to introduce a 22‐bp deletion, generating *Spink5* knockout mice. Histologically, these *Spink5* ^−/−^ mice exhibited complete SC detachment and absence of LEKTI in the skin [27] (Figure 3).

## Limitations of the *Spink5* ^−/−^ model and development of new murine models to tackle NS pathophysiology

Given that *Spink5* ^−/−^ mice die soon after birth, they cannot be used to test potential therapeutic compounds or to study the molecular pathology of NS during adulthood. Therefore, there is a pressing need for viable mouse NS models. To address this challenge, a mosaic *Spink5* ^−/−^ mouse was developed [28]. It is known that injection of TALENs into the cytoplasm of mouse zygotes can result in mosaicism in founder mice, due to activity of these nucleases during the two‐cell stage or even later. Therefore, the authors used this approach to select for animals that showed a desquamating phenotype in approximately 40% of their bodies. Importantly, lower doses of TALENs can generate mice with even milder phenotypes (with skin abnormalities in 10% of their bodies). In other words, the extent of mosaicism can be controlled by altering the dosage of TALENs. Sequencing of DNA isolated from cells obtained from lesional skin showed inactivating mutations in both *Spink5* alleles, while sequencing of DNA obtained from non‐lesional sites showed wild‐type *Spink5* alleles. A more robust procedure for reliable generation of mosaic mice involves genetic manipulation of the two‐cell embryo directly [29]. The advent of viable NS mouse models is expected to provide insights into the potential compensatory mechanisms that develop in many NS patients enabling them to survive the neonatal period to adulthood.

Another strategy to generate viable NS models involves the development of a conditionally inducible *Spink5* ^−/−^ mouse [30]. For this, *Lox*P sites were introduced flanking exon 3 of the *Spink5* gene to generate *Spink5* ^*fl/fl*^ mice, which were subsequently crossed with *KRT14‐CreERT2* ^*Tg/0*^ mice carrying a transgenic (*Tg*) cassette that drives tamoxifen‐inducible Cre recombinase expression under the keratin 14 promoter, active in basal keratinocytes. Deletion of *Spink5* in the epidermis was induced by intraperitoneal injection of tamoxifen or by topical application of 4‐hydroxytamoxifen for five consecutive days. These mice were viable and replicated the clinical features of NS, exhibiting high epidermal KLK activities, a defective epidermal barrier, abnormal epidermal differentiation, and skin inflammation. Importantly, the skin transcriptome of these *Spink5* ^*ie*−/−^ mice resembled the transcriptome of lesional skin of NS patients [30]. Furthermore, studies with these animals, supplemented with *in vitro* data, demonstrated that KLK7 can cleave IL‐36G to shorter peptides suggesting proteolytic activation of this cytokine, while KLK14 can cleave and degrade IL‐36A.

The NS epidermis exhibits a distinct IL‐36 signature [21, 30, 31]. Specifically, IL‐36A is detected at the mRNA level and not at the protein level. This discrepancy likely reflects the elevated activity of epidermal KLKs, including KLK14, which may degrade IL‐36A before it can accumulate [30]. Further experiments are needed to validate IL‐36 cleavage by KLK proteases *in vivo*, for which genetic mouse models could prove an invaluable tool.

An alternative approach to overcome the postnatal lethality limitations of *Spink5* ^−/−^ mice is to implant skin grafts, as demonstrated in the *Spink5* ^−/−^ and *Spink5* ^−/−^ *Par2* ^−/−^ models [32] using the skin flap technique [33]. In this procedure, the neonatal *Spink5* ^−/−^ mouse is immediately euthanized and its skin removed. Next, an adult nude mouse or a mouse with the same genetic background (to avoid graft rejection) is anaesthetized, and a Π‐like full skin section is made on its back. The section is opened and the newborn skin is grafted. The section is then closed to overlay the graft and sutured. After 3 weeks, the outer Π‐like section is removed to expose the graft. This method allows the study of pathogenic mechanisms later in life, i.e. in adulthood. In addition, skin grafting can be employed to test candidate therapeutics. For example, skin grafting has been used to assess the effectiveness of a vaseline‐based cream containing KLK7 and KLK5 for the treatment of hyperkeratosis in harlequin ichthyosis [34]. Similarly, the approach can be applied to *Spink5* ^−/−^ grafts to test new compounds designed for NS therapy.

## *Spink5* ^−/−^ mice reveal pharmacological targets for NS

Besides constitutive inflammation, the *Spink5* ^−/−^ epidermis exhibits abnormally elevated proteolysis, as revealed by *in situ* zymography. This is primarily due to unopposed activities of KLK proteases (KLK5, KLK7, and KLK14) but also other proteolytic enzymes present in LEKTI‐deficient epidermis, including elastase [35]. *In vitro* and *in vivo* studies have demonstrated that KLK5 acts as the initiator of the KLK cascade. Transgenic and knockout models have substantiated the original hypothesis based on *in vitro* studies, elucidated the underlying pathways, and enabled identification and preclinical validation of NS‐specific drug targets. These mouse models are therefore valuable tools for evaluating both the functional correlation between epidermal proteolysis and inflammation, and the specific contribution of inflammation to NS pathogenesis.

## Targeting epidermal proteases to revert or even rescue the NS lethal phenotype

Epidermal proteolysis executed by KLKs regulates the cleavage of corneodesmosomes and shedding of the SC layers. Based on *in vitro* experiments, KLK5 was hypothesized to activate PAR2 to drive the inflammatory reaction in the epidermis [13]. To this end, it should be mentioned that another protease, matriptase (also known as St14), which is also expressed in the epidermis, was previously considered to play an important role in desquamation, as *in vitro* proteolytic assays showed that it can enhance the activation of proKLK5 and proKLK7 [36]. Therefore, the first *in vivo* target validation study focused on St14. To address the potential role of St14 in regulating the epidermal KLK cascade *in vivo*, *Spink5* ^−/−^ *St14* ^−/−^ mice were generated [36]. If St14 were the principal activator of the KLK cascade, deletion of *St14* would be expected to rescue the NS phenotype. However, although *Spink5* ^−/−^ *St14* ^−/−^ mice appeared normal at birth, the authors did not provide evidence that these mice survived the neonatal phase. Furthermore, in human epidermis, St14 localizes in the SB and SS and not in the SC, where KLKs are active, arguing against the role of St14 in epidermal desquamation [37]. It should also be noted that *St14* ^−/−^ mice do not live longer than 48 h and die from severe epidermal defects [38].

*Spink5* ^−/−^ *Klk5* ^−/−^ mice were subsequently generated to examine the role of KLK5 in NS [39]. Deletion of *Klk5* in the *Spink5* ^−/−^ background reduced excessive epidermal desquamation and inflammation and rescued neonatal lethality. However, 70% of the mice survived for approximately 1 week, while the surviving 30% lived for up to 5 months [40, 41]. This suggested that, in the absence of Klk5, other proteases take over after the neonatal stage to drive excessive epidermal desquamation and inflammation evident around the end of the first week from birth. Rescue of neonatal lethality of *Spink5* ^−/−^ by deletion of *Klk5* was later verified by another group [26]. Taken together, these *in vivo* findings suggest that KLK5 has a dominant role over St14 in the regulation of epidermal desquamation.

The dominant role of KLK5 in the regulation of epidermal proteolysis is also supported by studies using *Spink5* ^−/−^ *Klk7* ^−/−^ mice, which die within a few hours after birth, similar to *Spink5* ^−/−^ mice. Nevertheless, deletion of both *Klk5* and *Klk7* fully rescued the NS phenotype to adulthood [26]. Finally, deletion of *Klk6*, encoding for Klk6 protease — also expressed in the epidermis and can autoactivate [42] — did not rescue the lethal phenotype of *Spink5* ^−/−^ mice [41, 43]. Detailed analysis of the *Klk5* ^−/−^ mouse phenotype suggests that targeting KLK5 is safe [44]. Collectively, these mouse model studies highlight the dominant role of KLK5 over other epidermal proteases in NS and validate a novel NS disease‐specific molecular target for pharmacological intervention.

Dual targeting of KLK5 and KLK7 with a bispecific antibody for treatment of AD and NS has recently been described [45]. This antibody is a heterodimer composed of one chain from anti‐KLK5 and one chain from anti‐KLK7 antibody. The pharmacodynamic and pharmacokinetic properties were studied in cynomolgus monkeys [46]. The bispecific surrogate against mouse Klk5 and Klk7 was tested in conditional tamoxifen‐induced *Spink5* ^−/−^ mice and was found to reduce the expression of proinflammatory cytokines and improve NS symptoms [47].

Importantly, the translational significance of these findings is underscored by ongoing clinical trials with KLK inhibitors. In particular, NCT05211830 (https://clinicaltrials.gov/search?term=NCT05211830%20) aims to administer a potent low molecular weight (LMW) inhibitor of KLK5, KLK7, and KLK14, formulated as a cream, in NS patients. NCT05979831 (https://clinicaltrials.gov/study/NCT05979831?term=NCT05979831) involves the administration of the DS‐2325a KLK5 inhibitor in a phase Ib/II trial to assess safety, efficacy, and pharmacokinetics in NS patients. In addition, clinical trials NCT06953466 (https://clinicaltrials.gov/study/NCT06953466?term=NCT06953466) and NCT05789056 (https://clinicaltrials.gov/study/NCT05789056?term=NCT05789056) aim to evaluate kallikrein inhibitor QRX003, as a lotion, in NS patients. No detailed data on the chemical specificity of QRX003 have been reported.

In the future, delineation of the putative role(s) of Klk13 and Klk14 proteases in NS should be investigated, since both proteases are abundantly expressed in the epidermis and have been implicated in the regulation of epidermal homeostasis and inflammatory responses. To address this, *Spink5* ^−/−^ *Klk13* ^−/−^ or *Spink5* ^−/−^ *Klk14* ^−/−^ double knockout mice should be generated. However, in the case that deletion of *Klk13* or *Klk14* does not rescue the phenotype (as found with *Spink5* ^−/−^ *Klk7* ^−/−^ mice), triple knockout mice such as *Spink5* ^−/−^ *Klk5* ^−/−^ *Klk13* ^−/−^ or *Spink5* ^−/−^ *Klk5* ^−/−^ *Klk14* ^−/−^ mice may be required. A major challenge in this effort is the fact that *Klk13* ^−/−^ and *Klk14* ^−/−^ mice are currently not available, as well as that *Klk5* ^−/−^ *Klk13* ^−/−^ and *Klk5* ^−/−^ *Klk14* ^−/−^ mice cannot be generated by crossing due to the close proximity of these corresponding *Klk* genes, therefore requiring a dual‐targeted genome‐editing strategy.

## Targeting the inflammatory phenotype of NS and/or excessive epidermal desquamation

NS patients display increased epidermal inflammation that is associated with erythema, itching, and reduced quality of life. In the epidermis, the major inflammatory pathways are mediated by protease‐activated receptor 2 (*Par2*), cathelicidin (*Camp*), Il‐36 (*IL36*), Il‐17A (*IL17A*), and Tnfα (*TNF*). Their effects and contributions to NS pathology have been preclinically validated using mouse models and in certain cases have been translated into clinical practice, as outlined below.

### The role of cathelicidin in NS

*In vitro*, KLK5 and KLK7 cleave the antimicrobial peptide precursor cathelicidin to produce LL‐37 and other shorter active antimicrobial peptides [48]. The generation of antimicrobial peptides has been demonstrated *in vivo* by subcutaneous injection of active KLK5 protease in a wild‐type mouse and analysis of epidermal extracts using surface‐enhanced laser desorption/ionization (SELDI)‐MS. As expected, KLK5 not only produced peptides derived by proteolytic processing of cathelicidin but also induced erythema and infiltration of inflammatory cells. In contrast, injection of KLK5 in the skin of *Camp* ^−/−^ mice resulted in a reduced inflammatory reaction [49]. Furthermore, *Spink5* ^−/−^ skin displayed increased expression of cathelicidin‐derived antimicrobial peptides with a profile resembling rosacea [49]. This study provided proof‐of‐principle for targeting KLK5 for the treatment of rosacea but also established the *Spink5* ^−/−^ mouse as a new model for rosacea. Gels containing azelaic acid are now used for the treatment of rosacea, and their action is mediated through inhibition of KLK5 and cathelicidin gene expression, and inhibition of epidermal serine proteolytic activity, as demonstrated in mice and in patients [50].

To provide further evidence for the functional implication of cathelicidin in the pathophysiology of NS, *Spink5* ^−/−^ *Camp* ^−/−^ mice were generated [40, 41]. Deletion of *Camp* on a *Spink5* ^−/−^ background failed to rescue the pathological excessive epidermal desquamation and associated neonatal lethality. However, *Spink5* ^−/−^ *Camp* ^−/−^ epidermis showed reduced expression of proinflammatory cytokines, such as *Tslp*, *Tnf*, *Il17a*, and *Il23a*. Deletion of both *Klk5* and *Camp* on a *Spink5* ^−/−^ background not only diminished epidermal inflammation and excessive epidermal desquamation at the neonatal stage but also rescued neonatal lethality, with *Spink5* ^−/−^ *Klk5* ^−/−^ *Camp* ^−/−^ mice living much longer than *Spink5* ^−/−^ *Klk5* ^−/−^ mice, namely, up to 1 year [40, 41].

### The role of *Tnf* in NS

The anti‐TNFα chimeric antibody infliximab showed beneficial effects and reduced epidermal inflammation when administered in two adult NS patients in two separate case studies [51, 52], and in one infant case report [53]. These findings, in combination with the established strongly upregulated expression of *Tnf* in the epidermis of *Spink5* ^−/−^ mice [39], provided the rationale for investigating the role of *Tnf* in NS. The generation of *Spink5* ^−/−^ *Tnf* ^−/−^ mice [41, 54] confirmed that deletion of *Tnf* alone in a *Spink5* ^−/−^ background is not sufficient to rescue neonatal lethality and severe NS pathology, despite diminished expression of epidermal proinflammatory cytokines, in accordance with the previous case reports. Anti‐TNFα therapy with infliximab was also recently reported to significantly reduce skin lesions in two adult NS patients [55]. Collectively, these studies provide strong support for the repurposing of anti‐TNFα therapeutics to treat the inflammatory manifestations of NS.

Since *Spink5* ^−/−^ *Klk5* ^−/−^ mice display delayed inflammation, triple knockout *Spink5* ^−/−^ *Klk5* ^−/−^ *Tnf* ^−/−^ mice were generated to determine whether a combination of TNFα targeting and KLK5 inhibition can rescue both epidermal inflammation and excessive epidermal desquamation. *Spink5* ^−/−^ *Klk5* ^−/−^ *Tnf* ^−/−^ mice were found to live normally to late adulthood; therefore, cocktails of KLK5 inhibitors and anti‐TNFα biologics were proposed as the first disease‐specific treatment for NS [54]. These findings also indicated that Tnfα drives the delayed inflammatory phenotype in *Spink5* ^−/−^ *Klk5* ^−/−^ mice. Currently, we are close to clinical implementation of these findings, since several anti‐TNFα biological drugs are already approved and widely prescribed in clinical practice, while KLK5 inhibitors are under preclinical or clinical evaluation, as outlined below.

### The role of *Par2* in NS

PAR2 is a key receptor mediating inflammatory responses. Previous *in vitro* experiments showed that KLK5 activates PAR2 to drive NS inflammation and TSLP production [13]. Notably, the high concentration of active KLK5 used has not been reported *in vivo*. To delineate the role of PAR2 in NS *in vivo*, *Spink5* ^−/−^ *Par2* ^−/−^ mice were generated [32]. These mice displayed neonatal lethality associated with severe desquamation, similar to *Spink5* ^−/−^ mice. *Spink5* ^−/−^ *Par2* ^−/−^ mice also showed reduced *Tslp* expression, although this was observed only at the neonatal stage. In skin grafts, *Spink5* ^−/−^ *Par2* ^−/−^ skin showed increased *Tslp* expression to levels comparable to *Spink5* ^−/−^ grafts.

The minimal contribution of Par2 signalling in driving epidermal inflammation in NS was corroborated by Zhu *et al*, who demonstrated that KLK5 induces atopic symptoms independent of PAR2 activation [56]. It is possible that Par2 is activated by proteases present in the environment, such as dust mite proteases that enter the body through a defective epidermal barrier [57]. Conversely, endogenously expressed proteases, such as prostasin/Prss8, may be involved in PAR2 activation, as demonstrated in transgenic mouse models [58]. The potential involvement of prostasin/Prss8 in NS has not been studied and warrants future investigation.

### Effect of nuclear factor (erythroid‐derived 2)‐like 2 (Nfe2l2 or Nrf2) activation in NS

Nrf2 is the master regulator of antioxidant defence and has been functionally linked with psoriasis [59]. Dimethyl fumarate (DMF), a compound that acts as a strong upregulator of the Nrf2 pathway, has been approved in Germany under the tradename Fumaderm® for the treatment of psoriasis [60]. The restricted expression of constitutively active Nrf2 (caNrf2) in epidermal keratinocytes of a transgenic mouse (*K5Cre CMV‐caNrf2*) resulted in Nrf2 expression levels comparable to those achieved through pharmacological activation. This transgenic mouse showed hyperkeratosis, acanthosis, and mild epidermal inflammation. Hyperkeratosis is likely associated with strong upregulation of the secretory leukocyte protease inhibitor (Slpi), an endogenous inhibitor of KLK7 [61]. Previously, activation of Nrf2 *in utero* was shown to stabilize the epidermal barrier in loricrin‐knockout mice [62]. These findings provided the rationale for testing the potential therapeutic effect of targeting Nrf2 in NS, which has a markedly defective epidermal barrier.

**Figure 4:**
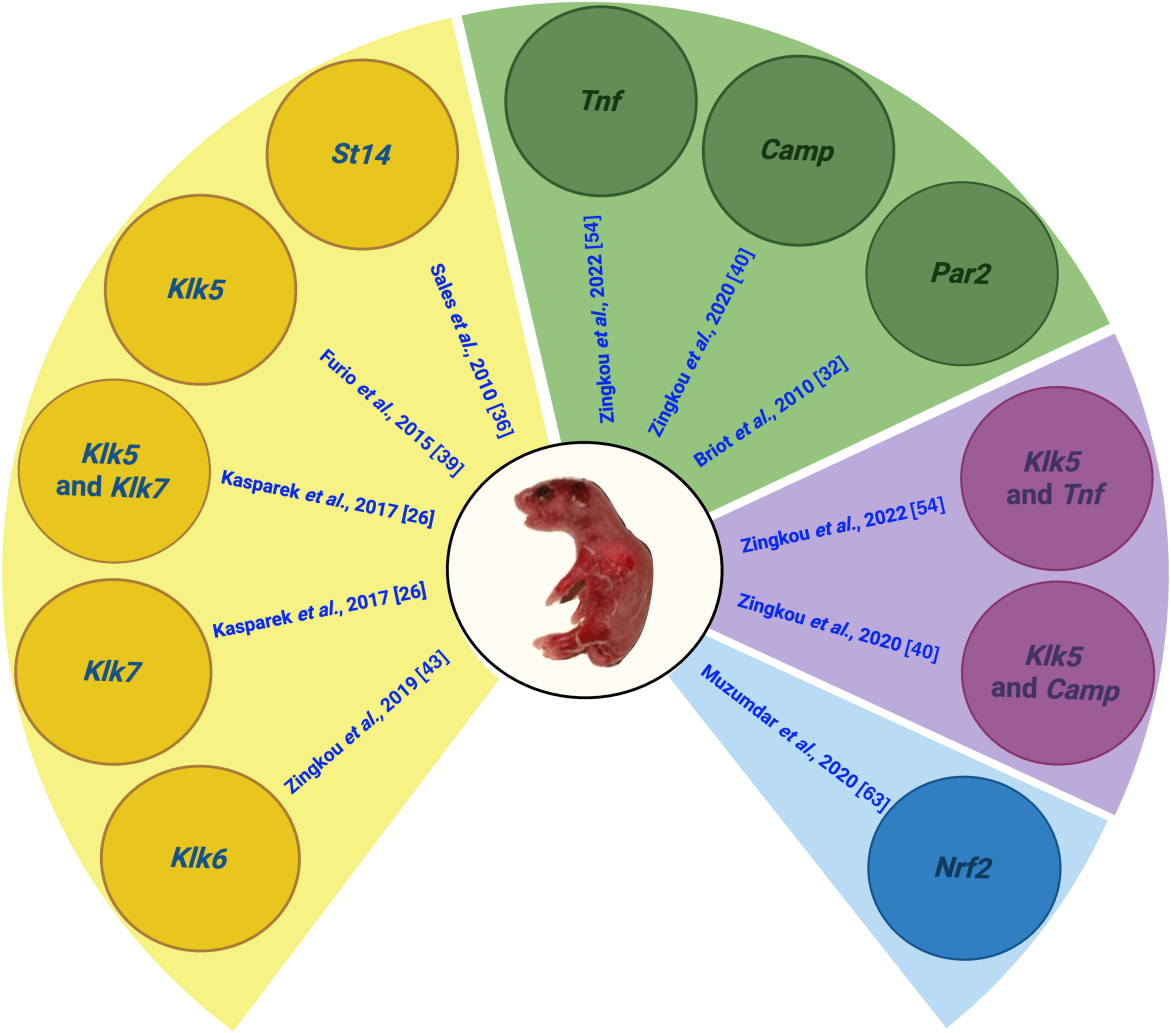
Overview of new NS mouse models that are based on the *Spink5* ^−/−^ mouse. These mice have greatly assisted in the delineation of the molecular pathology of NS and in the identification of new therapeutic targets. Created in BioRender. Zingkou E (2025) https://BioRender.com/jo0mget

Transgenic expression of a constitutively active Nrf2 (caNrf2) in keratinocytes of *Spink5* ^−/−^ mice normalized their macroscopic appearance and whisker structure, promoted attachment of the SC, restored the epidermal barrier, and reduced the expression of proinflammatory cytokines, including *Tnf* and *Tslp*. However, the authors did not report any data on the viability of *Spink5* ^−/−^ *Nrf2* ^*tg/tg*^ mice [63]. Overall, this study suggested that agents activating Nrf2 may have therapeutic potential in NS. One such agent is the already approved drug dimethyl fumarate, which may be worth evaluating in NS. Figure 4 summarizes all the above‐mentioned models.

### Other proinflammatory cytokines in NS

TSLP is considered a key proinflammatory cytokine that drives not only inflammatory reactions but also pruritus, and intradermal injection of TLSP in mice induces pruritus [64]. Its potential role in NS remains to be determined. Notably, *Tslp* ^−/−^ mice are available [65] and may be crossed with viable *Spink5* ^*+/−*^ mice. In addition, new anti‐TSLP therapeutics have been developed, such as the monoclonal antibody tezepelumab, which was approved by the FDA in 2011 for the treatment of asthma [66]. Repurposing anti‐TSLP therapies to treat inflammatory manifestations in NS may be worth consideration, and new murine models, such as the *Spink5* ^−/−^ *Tslp* ^−/−^, are valuable tools for generating the preclinical data required to support or challenge the repurposing of these therapies for NS.

Previous studies have shown increased Il‐17 [39] and Il‐36 in animal models of NS [30], suggesting that these proinflammatory cytokines may be key players in NS. To investigate their roles in NS pathophysiology, *Spink5* ^−/−^ *Il36r* ^−/−^ and *Spink5* ^−/−^ *Il17ra* ^−/−^ mice should be generated, as both *Il36r* ^−/−^ [67] and *Il17ra* ^−/−^ [68] mice are already available and can be crossed with viable *Spink5* ^*+/−*^ mice. The *Il17ra* ^−/−^ mice carry a deletion in the Il‐17 receptor subunit A, which is required for formation of the heterodimer functional Il‐17 receptor with other members of the Il‐17 receptor family. Since IL‐36 also seems to be implicated in NS [30, 31], anti‐IL‐36 therapeutics could be tested as alternative therapies for NS patients. Currently available anti‐IL‐36 biologics include spesolimab, an IL‐36 receptor antibody, approved by the FDA in 2022 for the treatment of generalized pustular psoriasis (GPP) [69], and imciromab, another anti‐IL‐36R antibody, which has completed phase II trials for GPP, demonstrating promising results [70].

## Transgenic models: viable tools for preclinical testing of pharmaceutical compounds

Transgenic mouse models have been generated as useful tools in studies aiming to delineate the pathological mechanisms underlying NS, and to test new potential pharmacological compounds. Furthermore, studies using transgenic mouse models complement those studies performed with knockout mice.

### The *KLK5* transgenic mouse model

This model carries the transgene encoding for the human *KLK5* gene under the control of the involucrin promoter that is active in the upper SS and SG layers. In contrast to *Spink5* ^−/−^, these mice recapitulate NS symptoms, while they provide a viable model, as they live for more than a year. *Tg*‐*KLK5* mice exhibit lower body size and weight, increased epidermal inflammation, itching, and desquamation [71]. Epidermal overexpression of *KLK5* in transgenic mice induced the synthesis of KLK5 and allows the testing of KLK5 inhibitors developed as candidate drugs based on the structure of the human orthologue. In this context, a new borocyclic compound, GSK951, developed by GlaxoSmithKline, was applied as a topical cream on the epidermis of *Tg‐KLK5* mice and was found to reduce transepidermal water loss and the expression of proinflammatory cytokines [72].

### The *KLK7* transgenic mouse model

This model carries the transgene encoding for the human *KLK7* gene under control of the early promoter of the SV40 virus (SV40e) [73]. *Tg‐KLK7* mice displayed increased epidermal thickness and inflammation, itching, and hyperkeratosis in a manner that resembles AD. Their pathological phenotype is less severe than that of *Tg‐KLK5* mice, further supporting the dominant role of KLK5 over other KLKs as the initiator of the epidermal proteolytic cascade. These mice serve as a model for AD, in contrast to *Tg‐KLK5* mice that serve as a model for NS. In fact, a patent has been disclosed for new heterocyclic KLK7 inhibitors that were tested on this mouse model as new pharmacological agents for the treatment of AD [74].

### The *KLK14* transgenic mouse model

The *Tg*‐*KLK14* transgenic mouse carries the human *KLK14* transgene under the control of the involucrin promoter (*Tg‐KLK14*). *Tg‐KLK14* mice showed severe hair abnormalities [31]. Specifically, in *Tg‐KLK14* mice, the hairs did not grow, and the mice showed hyperplastic hair follicles and curled ingrowth hair shaft in the anagen hair follicle. However, on day 50, *KLK14* expression was lost, and the mice showed normal hair growth, albeit the hair was sparse. This suggests that overactivation of KLK14, at least partially, underlies the hair defects found in NS. Further, *Tg‐KLK14* mice showed increased IL‐36‐associated epidermal inflammation, something that has also been found in *Spink5* ^−/−^ mice [30]. Collectively, these findings suggest that IL‐36 signalling could be a new pathway for pharmacological intervention in NS and support the repurposing of spesolimab for the treatment of NS.

### The *Klk6* transgenic mouse model

Tg‐*Klk6* transgenic mice were generated in which the mouse *Klk6* transgene is under the control of the tetracycline transactivator (Tet^off^) promoter. These Tg‐*Klk6* mice were crossed with mice bearing the tetracycline transactivator under the control of the keratin 5 promoter [75]. Tg‐*Klk6* mice highly overexpress *Klk6* in the epidermis and show psoriasis‐like symptoms mediated by Par1. Tg‐*Klk6* mice also developed inflammatory joint disease, providing direct evidence that skin inflammation alone is sufficient to drive joint disease. Epidermal levels of *Il17a*, *Il17f*, *Il36a*, and *Klk13* were highly elevated in Tg‐*Klk6* mice, with all showing more than 200‐fold increases compared with healthy mouse skin. Some cutaneous symptoms exhibited by Tg‐*Klk6* mice resemble those seen in NS and, recently, it was shown that lesional NS skin displays elevated KLK6 and KLK13 expression [76].

Table 1 summarizes representative murine models for NS used to identify molecular mechanisms of NS, with potential for clinical translation. One challenge in extrapolating observations from a transgenic mouse overexpressing an epidermal protease to the human disease phenotype is that transgene expression levels may be abnormally high compared with the levels found in patients, potentially creating an artificial phenotype. Therefore, caution should be taken when translating study outcomes with transgenic mice to clinical phenotypes. In this context, co‐evaluation of results from knockout mice may facilitate understanding of disease pathways and the selection of appropriate drug target and putative secondary targets. The study of NS represents a prototype for this approach, as it has been successfully implemented through collective evaluation of the mouse model studies described above.

**Table 1:** Mouse models employed for validation of novel targets with translational applications.

Animal model	Phenotype	Clinical translation/support/suggestion
*Spink5* ^−/−^ *Klk5* ^−/−^	Rescue of neonatal lethality, delayed inflammation, and excessive epidermal desquamation	Validation of the KLK5 target for NS NCT05211830, NCT06953466, NCT05789056
*Spink5* ^−/−^ *Tnf* ^−/−^	Reduction of epidermal inflammation	Infliximab treatment of patients Supports clinical utility
*Spink5* ^−/−^ *Camp* ^−/−^	Reduction of epidermal inflammation	Supports the use of azelaic acid creams or other compounds that silence *Camp*
*Spink5* ^−/−^ *Nrf2* ^*tg/tg*^	Reduction of epidermal inflammation	Supports the use of drugs that upregulate Nrf2 (e.g. DMF)
*Tg*‐*KLK5*	Netherton syndrome	Drug testing: development of a new KLK5 inhibitor (GSK951)

## Mouse models versus organotypic cultures

**Figure 5:**
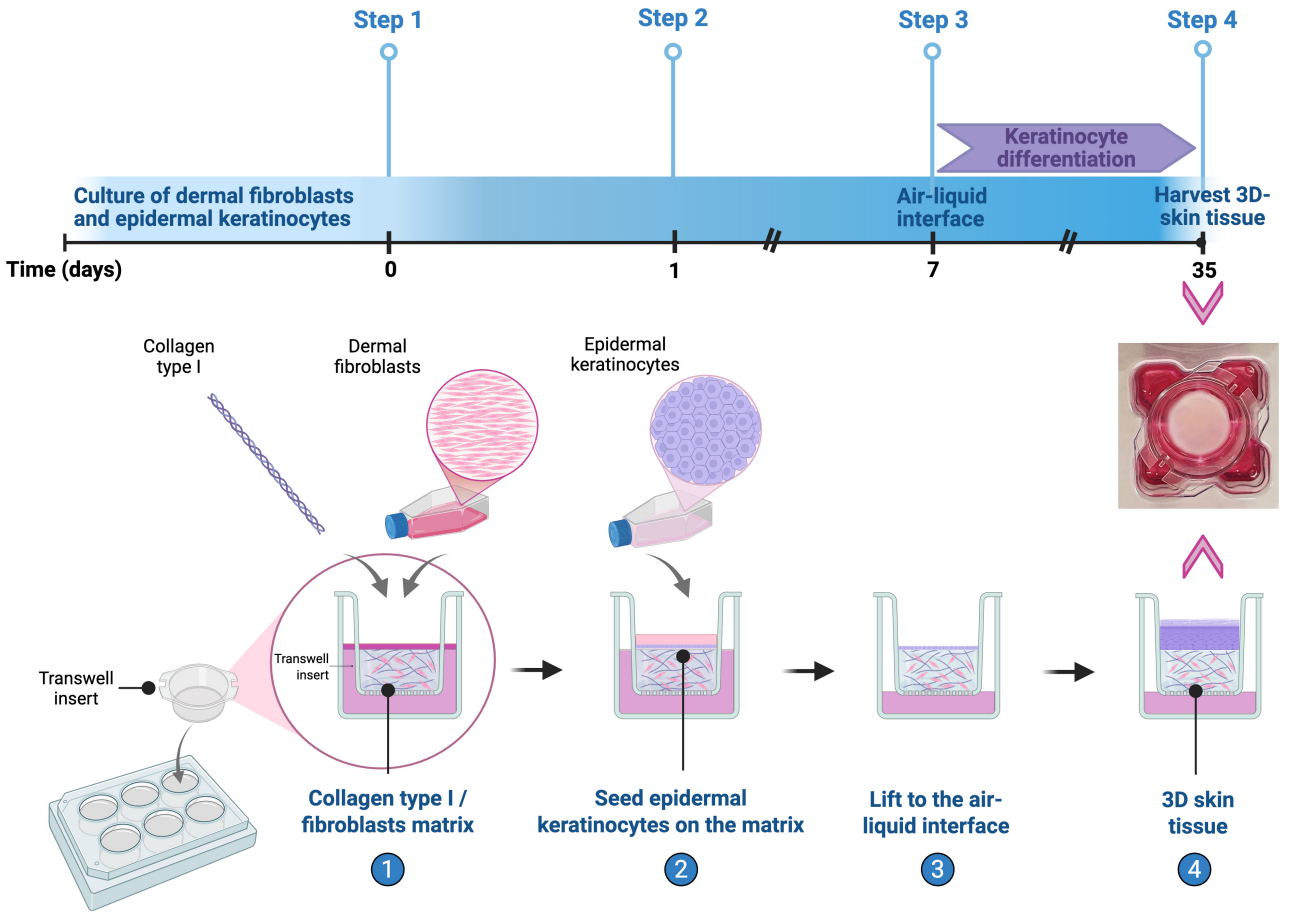
Schematic diagram of the process for the generation of a 3D organotypic culture. Timeline of *in vitro* engineering of 3D human skin equivalent and schematic overview of the development of 3D reconstructed human skin. Further, a macroscopic top view of the developed 3D human skin equivalent is presented. Created in BioRender. Zingkou E (2025) https://BioRender.com/m5ot9kx

The study of mice has certain disadvantages, including ethical concerns and ongoing efforts to reduce the number of experimental animals, as well as the need for lengthy experiments and increased costs. To reduce the time required to breed and cross animals, and avoid ethical issues, organotypic skin cultures have been developed. Moreover, organotypic skin cultures are ideal for screening potential therapeutic compounds by simulating human skin diseases [77] (Figure 5).

An organotypic skin model was generated using human keratinocytes in which *SPINK5* was knocked down with siRNA technology. This *in vitro* model recapitulated the NS phenotype and exhibited the typical skin barrier defect of NS – extensive SC detachment. Knockdown of *KLK7* or *KLK5* expression in these cells partially reverted the NS phenotype, with the effect of KLK7 siRNA being more pronounced compared with suppression of *KLK5* [78]. The same model has been generated independently by another group and showed the same results [79], which are in direct contrast with *in vivo* data from *Spink5* ^−/−^ *Klk5* ^−/−^ and *Spink5* ^−/−^ *Klk7* ^−/−^ mice [26, 39]. This is an example of *in vitro* data being in discrepancy with results from *in vivo* studies, underscoring the need to interpret *in vitro* study results with caution.

However, in a recent study, dual knockdown of *KLK5* and *KLK7* in shRNA‐mediated *SPINK5* silencing in keratinocytes or dual chemical inhibition completely reverted the NS phenotype [79]. The organotypic NS skin model based on siRNA‐mediated knockdown of *SPINK5* has been used to test potential inhibitors of KLK5 and KLK7 proteases for NS. Nevertheless, the findings should be carefully evaluated, since this model does not fully recapitulate the molecular pathology of NS. It is possible that residual expression of *SPINK5* alters the NS phenotype in this model. To address this, an organotypic NS model was developed by CRISPR–Cas9‐mediated elimination of *SPINK5* in human keratinocytes [80]. These cells were used as a model for NS therapy by *SPINK5* gene delivery. The assessment of *SPINK5* delivery on the normalization of the NS phenotype was performed after grafting genetically engineered organotypic cultures in nude mice. This gene delivery approach resulted in the recovery of 61.3% of LEKTI expression that translated to normalization of the epidermal architecture [80]. Whether targeting *KLK5* or *KLK7* in the *SPINK5* ^−/−^ organotypic skin model rescues the NS phenotype and recapitulates the *in vivo* conditions reported in mice remains to be determined.

Another factor that may account for discrepancies between organotypic models and mouse models is the fact that organotypic cultures typically contain only one or two cell types, namely keratinocytes in epidermis models or keratinocytes grown on fibroblasts in skin models. Consequently, other cell types, such as immune cells, do not contribute as they do *in vivo*. The compromised phenotype of organotypic models is further illustrated by previous studies in which *SPINK5* silencing [78, 79] or *SPINK5* ^−/−^ [80] resulted in epidermis that was thinner or at least had the same thickness as the epidermis derived from wild‐type keratinocytes, in direct contrast to the NS patient epidermis or the *Spink5* ^−/−^ mouse epidermis. Moreover, organotypic skin does not display papillomatosis of the epidermal layer, although it is a typical feature of NS.

Other problems with organotypic skin cultures are the inefficient diffusion of nutrients and their limited lifespan. These may be overcome by organotypic skin grafting. In this direction, an analogous study was performed where the long‐term effects of *ex vivo* gene therapy focusing on restoration of LEKTI expression in human NS keratinocytes were investigated. Briefly, lentiviral delivery of *SPINK5* in keratinocytes isolated from NS patients resulted in re‐expression of LEKTI. Three‐dimensional (3D) cultures generated with these keratinocytes did not show the NS‐associated abnormalities [81]. When these 3D cultures were grafted in nude mice, normalization of the grafted epidermis was observed [81]. This study provided a proof‐of‐concept for the efficacy of *ex vivo* gene therapy in NS and highlights a promising approach for the development of personalized therapies for NS patients.

## New directions from case reports

As mentioned above, most *in vivo* studies are corroborated by relevant case reports involving the administration of biological drugs in NS patients [82]. Here, we briefly present the clinical findings. Biological therapies for paediatric or adult NS patients have focused on the administration of secukinumab, an anti‐IL‐17A human monoclonal antibody; ixekizumab, an anti‐IL17A humanized monoclonal antibody; dupilumab, an anti‐IL‐4 human monoclonal antibody; anakinra, a recombinant IL‐1 receptor antagonist; infliximab, an anti‐TNFα chimeric monoclonal antibody; and ustekinumab, an anti‐p40 subunit of the IL‐12/IL‐23 axis targeting human monoclonal antibody.

Secukinumab has been preferentially administered to NS patients due to extensive studies implicating IL‐17A signalling in NS [83]. Among 11 reported cases of NS patients treated with secukinumab, eight showed improvement in cutaneous manifestations, such as erythema and scaling [84, 85, 86, 87, 88]. A clinical trial (NCT03041038) assessed the role of secukinumab in various types of ichthyosis, including three NS patients. By the primary endpoint at week 16, no significant improvement was noted. Nevertheless, two NS patients continued treatment and by week 32, reductions in itching, erythema, and scaling were reported [89]. Combination therapy with dupilumab and secukinumab yielded a positive response in a paediatric NS patient [90]. Similarly, a significant improvement in itching, scaling, and erythema was observed in seven NS patients after 24 weeks of this combination treatment. In some of these patients, administration of secukinumab alone did not show a reduction of itching [91]. In another study, five NS patients were treated with intravenous immunoglobulins, ixekizumab, dupilumab, and anakinra, alone or in combination. Despite initial improvements, symptoms later exacerbated leading to discontinuation of therapy, except for one patient treated with ixekizumab [92]. Collectively, these clinical reports underscore the need for further mouse studies using new models, such as the *Spink5* ^−/−^ *Il17r* ^−/−^ mouse model. Molecular analyses of *Spink5* ^−/−^ *Il17r* ^−/−^ mice versus *Spink5* ^−/−^ *Tnf* ^−/−^ mice, together with results from clinical case studies, will likely determine whether targeting IL‐17A will yield more effective therapies for NS than anti‐TNFα therapeutics.

## Conclusions

Collectively, these studies have identified and validated KLK5 as a key target for the treatment of NS and justify ongoing efforts to develop specific KLK5 inhibitors for clinical evaluation. The safety of KLK5 targeting has been highlighted by the lack of toxicity observed in *Klk5* ^−/−^ mice, which are indistinguishable from wild‐type animals, reproduce normally, and thrive throughout a normal lifespan [42]. Various compounds belonging to different chemical groups have been studied, such as phenolic amidine‐based inhibitors [93], coumarin‐based suicide inhibitors [94], peptide‐based inhibitors [95, 96], and compounds containing imidazole and benzamidine moieties [97]. The most promising KLK5 inhibitors under development appear to be borocyclic‐based compounds [98], with one of these compounds, named GSK951, preclinically validated in *Tg‐KLK5* [72].

The preclinical efficacy of dual targeting of KLK5 and KLK7 proteases, as well as both KLK5 protease and TNFα signalling, has been shown using *Spink5* ^−/−^ *Klk5* ^−/−^ *Klk7* ^−/−^ [26] and *Spink5* ^−/−^ *Klk5* ^−/−^ *Tnf* ^−/−^ mice [54]. Epicutaneous application of a synthetic phosphonate‐based KLK7 inhibitor on the epidermis of 3‐day‐old *Spink5* ^−/−^ *Klk5* ^−/−^ mice led to a significant reduction in exfoliation and suppressed the production of proinflammatory cytokines, further confirming the necessity of targeting both KLK5 and KLK7 [99]. Notably, the phosphonate activity‐based probe (ABP) for KLK7 acts as a suicide inhibitor and reduces exfoliation in *Spink5* ^−/−^ *Klk5* ^−/−^ mice [99], supporting the concept that ABPs can act both as diagnostic and as therapeutic agents (theranostics) [100, 101].

On the other hand, the markedly beneficial effects of targeting the TNFα pathway observed in mice support the use of anti‐TNFα biologics in the clinical management of NS, with the added value that several anti‐TNFα monoclonal antibodies and chimeric proteins are currently approved and widely prescribed for various inflammatory diseases. Notably, studies of these mouse models may reveal new unexplored avenues for pharmacological intervention of NS, such as the IL‐36 proinflammatory pathway, for which approved drugs are currently available.

## Author contributions statement

EZ, GP and GS conceived the study. EZ, EB, GP and GS developed the methodology. EZ, EB, GP and GS conducted the formal analysis. EZ, GP and GS wrote the original draft of the manuscript. EZ, GP and GS were responsible for project administration. GS and GP acquired funding for the study.

## Data Availability

All data are described in the article.
